# Enhanced recovery after surgery for laparoscopic gastrectomy in gastric cancer: A prospective study

**DOI:** 10.1097/MD.0000000000024267

**Published:** 2021-02-19

**Authors:** Yanrui Liang, Hao Liu, Li Zhen Nurse, Yu Zhu, Mingli Zhao, Yanfeng Hu, Jiang Yu, Cai Li, Kexuan Liu, Guoxin Li

**Affiliations:** a Department of General Surgery; b Department of Anesthesiology, Nanfang Hospital, Southern Medical University, Guangzhou, Guangdong, China.

**Keywords:** enhanced recovery after surgery, gastric cancer, laparoscopic distal gastrectomy, post-operative complication, post-operative hospital stay

## Abstract

**Background:**

Laparoscopic distal gastrectomy (LDG) has been highlighted for its safety and better short-term clinical outcomes in treating gastric cancer. However, only a slight reduction of the post-operative hospital stay was observed in gastric cancer patients undergoing LDG with conventional perioperative management, compared to patients undergoing open surgery. Thus, an enhanced recovery after surgery (ERAS) program for LDG is needed to further reduce the post-operative hospital stays. This prospective, open-label, single-arm cohort study aimed to assess the safety and efficacy of the ERAS program for gastric cancer patients undergoing LDG.

**Material and Methods:**

All patients with gastric cancer indicated for LDG were consecutively enrolled from December 2016 to January 2018. The ERAS program included short fasting time, effective perioperative pain management, early, goal-oriented ambulation, and oral feeding. The safety assessment was the incidence of post-operative complications, mortality, and readmission in 30 days. The primary efficacy assessment was recovery time defined by post-operative hospital stays and rehabilitative rate on post-operative day 4.

**Results:**

Ninety-eight of 114 patients were finally enrolled. The incidence of post-operative complication, mortality, and readmission in 30 days was 20. 4%, 0%, 7.1%, respectively. The Clavien-Dindo grade III complication rate was 6.1%, while the pulmonary complication rate was 1% only. The median post-operative stay was 6 days (5.0-7.0 days), and the rehabilitative rate on post-operative day 4 was 78%.

**Conclusions:**

The ERAS program might be optimal perioperative management for gastric cancer patients after LDG without compromising safety.

**Trial number:**

NCT03016026

## 1 Introduction

Radical surgery is the cornerstone in treating resectable gastric cancer and contributes to an improved survival rate for gastric cancer patients.^[1–3]^ Nowadays, the interest has been turned to minimally invasive surgery including stomach function preservation and life quality improvement while maintaining surgical curability. Since the 1980s, the technique of laparoscopic surgery has become frequently applied for a wide field of indications for its safety and efficacy.^[4]^ In Eastern countries, Laparoscopic distal gastrectomy (LDG) has been highlighted for its safety and better short-term clinical outcomes.^[2,5–7]^ By applying the laparoscopic technique, we can minimize surgical insults and maximize the quality of life without sacrificing radicality and survival. However, based on the findings of the multicenter randomized clinical trials from the Chinese Laparoscopic Gastrointestinal Surgery Study-01 (CLASS-01) and the Korean Laparo-endoscopic Gastrointestinal Surgery Study-01, the reduction of the post-operative hospital stay of the LDG group was fewer than 1 day, compared with the open surgery group.^[6,8]^ Thus, the conventional perioperative management program for LDG might need to be optimized.

Enhanced Recovery After Surgery (ERAS), also known as Fast-Track, was first applied in a project to improve the outcomes of coronary artery bypass surgery in 1994.^[9]^ With years of development by testing protocols, running symposiums, and involving national health ministries, the ERAS Society has developed a perioperative practice model to enhance recovery for patients undergoing gastrointestinal surgery including gastrectomy and colorectal surgery.^[10–13]^ Their protocols use multimodal approaches to perioperative care including short fasting time, intravenous fluid restriction, early oral feeding, immediate mobilization, and appropriate analgesia. However, whether and which ERAS program is the optimal perioperative care for LDG for gastric cancer remains unknown. In this study, we aimed to assess the safety and efficacy of the established ERAS program for LDG in patients with gastric cancer.

## 2 Material and methods

### 2.1 Patients

This study was a prospective, open-label, single-arm cohort study with a follow-up period of 30 days, conducted in Nanfang Hospital, Southern Medical University (Guangzhou, China). Patients with gastric cancer were assessed for eligibility from December 2016 to January 2018.^[14]^

The inclusion criteria were age from 18 to 75 years, pathologically proven primary gastric adenocarcinoma by endoscopic biopsy, a clinical tumor that penetrated the visceral peritoneum (cT4a) or lower T factor, with or without lymphatic metastasis (N0-N3), no metastasis (M0), expected curative resection through LDG, no history of prior or other malignancies within the past 5 years prior to enrollment with the exception of basal cell carcinoma, Eastern Cooperative Oncology Group performance status of grade 0 or 1,^[15]^ American Society of Anesthesiologists (ASA) physical status of grade I or II,^[16]^ adequate organ functions and written informed consent. The clinical TNM classification of gastric cancer was based on the results of gastroscopy, thoracic, abdominal computed tomography scans, and the American Joint Committee on Cancer Cancer Staging Manual Seventh Edition.^[17]^

The exclusion criteria were severe mental disorder, women of childbearing potential who were pregnant or breastfeeding, history of previous upper abdominal surgery, history of previous neoadjuvant chemotherapy, radiotherapy or clinical trial treatment within 3 months, history of other malignant diseases within the past 5 years, contraindications for surgery including active systemic infections, coagulation disorders, other major medical illnesses of the cardiovascular, respiratory, or immune system, history of myocardial infarction or cerebrovascular accident within past 6 months, and emergency surgery due to complications (bleeding, obstruction or perforation) that caused by gastric cancer. The withdrawal criteria were distant metastasis, curative resection through total gastrectomy, conversion to open surgery, combined organ resection, inability to undergo surgery or anesthesia for the changing illness state, intraoperative bleeding over 400 mL or transfusion, and required to withdraw by patients. The protocol of this study was approved by the Ethics Committee of Southern Medical University and registered at http://clinicaltrials.gov. The trial number was NCT03016026 (Supplemental Digital Content).

### 2.2 Procedures

Surgeons who met the following criteria were selected: performed at least 50 distal gastrectomies with D2 lymphadenectomy using open and laparoscopic approaches, annually performed at least 300 gastrectomies for patients with advanced gastric cancer. LDG was performed with either D1 plus or D2 lymphadenectomy. Reconstruction was performed by Roux-en-Y gastrojejunostomy or intracorporeal gastroduodenostomy. LN examination approaches followed our previous reports.^[18]^

### 2.3 ERAS program

Combining the recommendation of ERAS society and our clinical practice, we optimized the ERAS protocol.^[10]^ After the patient was admitted, preoperative counseling and education about the ERAS program were provided in the ward orally by a multidisciplinary team which consisted of the surgeon, anesthetist, and nurse. The education goal was to facilitate patients to understand the ERAS protocol and overcome the fear of surgery. We would initiate a nutritional supplement protocol for 5 to 7 days if the patient's Nutrition Risk Screening score was >3. In addition, we would start breathing training immediately after the patient's admission to reduce respiratory complications. Mechanical bowel preparation was not used before surgery. Patients were allowed to maintain normal oral diets until midnight and the intake of carbohydrate solution up to 2 hours before surgery. Prophylactic antibiotics were used during surgery according to local guidelines. In principle, no prophylactic antibiotics were used after surgery unless clear evidence of bacterial infections was shown. Multimodal analgesia consists of non-steroidal anti-inflammatory drugs before the induction of anesthesia, and surgical site infiltration was applied.^[14]^ Intermittent pneumatic compression was used as thrombosis prophylaxis. Surgeons adhered to the minimal invasion principle by using laparoscopic technique and ensuring incision less than 7 cm. Patients received an intravenous injection of 40 mg parecoxib sodium as post-operative pain management protocol. Patients started progressive oral feeding and goal-oriented ambulation in post-operative days 1 to 4 (PODs 1–4). Patients measured the distance of ambulation by the marker in the ward and reported it to the study nurse. The ERAS tube management principles consisted of no nasogastric tube or removing nasogastric tube within 6 hours after surgery, removing the urinary catheter and electrocardiographic monitoring within 24 hours after surgery, and no more than 1 drain and removing the drain within 72 hours after surgery. Rehabilitative and discharge criteria were defined as the following,

(1) no physical and psychological complaints,
(2) no intravenous therapy,
(3) no complications,
(4) tolerance of semi-fluid diet,
(5) safe ambulation of more than 1500 m, and
(6) Visual Analogue Scale (VAS) score <3 (Table 1).

**Table 1:** The ERAS program.

Time	Protocol
After admission	Preoperative counseling and education about the ERAS protocolInitiating a nutritional supplement protocol while NRS^∗^ score>3Breathing training
Preoperative	Written, informed consentNo bowel preparation and nasogastric tubeMaintain a normal diet until midnightOral carbohydrate solution (OS-1000 ml, Carbohydrate 10%) up to 2h before surgery
Day of surgery	Urinary catheter insertion after anesthesiaLaparoscopic distal gastrectomy and incision<7 cmMultimodal analgesia with surgical site infiltration of NSAIDs^†^Restricted fluid therapyInfusion warmer and warming blanketIntermittent pneumatic compression, anti-embolic stockingsExercise in bedSOW^‡^ after patient awakedUrinary catheter removal within 6 h after surgeryIn principle, no nasogastric tube insertion or removing nasogastric tube within 6 hNo more than 1 drain
POD-1^§^	SOW, if tolerableAmbulation with assistance, if tolerable (500–1000 m)Routine thrombosis prophylaxisRoutine analgesics including NSAIDsLaboratory examination
POD-2	FFD^||^, if tolerableAmbulation with assistance, if tolerable (1000–1500 m)Routine thrombosis prophylaxisRoutine analgesics including NSAIDs
POD-3	SFD^¶^, if tolerableAmbulation with assistance, if tolerable (>1500 m)Routine thrombosis prophylaxisRoutine analgesics including NSAIDsDrain removalLaboratory and imaging examination
POD-4	SBD^#^, if tolerableAmbulation, if tolerable (>1500 m)Check discharge criteriaDischarge recommended, if possible

### 2.4 Clinical parameters assessment

The multidisciplinary expert team evaluated all patients throughout the study. Demographic data and surgery-related data, including preoperative, operative, and pathological parameters, were obtained. Preoperative parameters included Eastern Cooperative Oncology Group performance status^[15]^ and ASA physical status.^[16]^ Operative parameters were the operation time and intraoperative blood loss. Pathological parameters were tumor size, retrieved lymph node, and pathological stage.^[19]^

### 2.5 Safety assessments

The incidence of post-operative complications, mortality, and readmission in 30 days was determined. Evaluation of perioperative complications was based on the Clavien-Dindo classification of surgical complications.^[20]^ Grade 1 indicated no need for pharmacological and surgical treatment. Grade 2 required pharmacological treatment and total parenteral nutrition. Grade 3a required radiological endoscopic or surgical intervention without general anesthesia. Grade 3b required radiological endoscopic or surgical intervention with general anesthesia. Grade 4 indicated a patient suffering a life-threatening complication, and Grade 5 was the death of the patient. The terminology of complications was in accordance with the Common Terminology Criteria for Adverse Events version 5.0.^[21]^

### 2.6 Efficacy assessments

The primary efficacy assessment was recovery time defined as post-operative stays and rehabilitative rate on POD-4. The secondary efficacy assessments were short-term recovery outcomes including first ambulation time, first flatus time, oral liquid diet recovery time, oral semi-fluid diet recovery time, drainage removal time, VAS score on PODs 0–3, and the compliance rate for the ERAS program.

### 2.7 Statistical analysis

For continuous variables, the data were presented as mean and median (interquartile range). For categorical variables, the data were presented as frequencies and percentages. SPSS 9.2 was used for these statistical analyses.

## 3 Results

### 3.1 Patients

**Figure 1:**
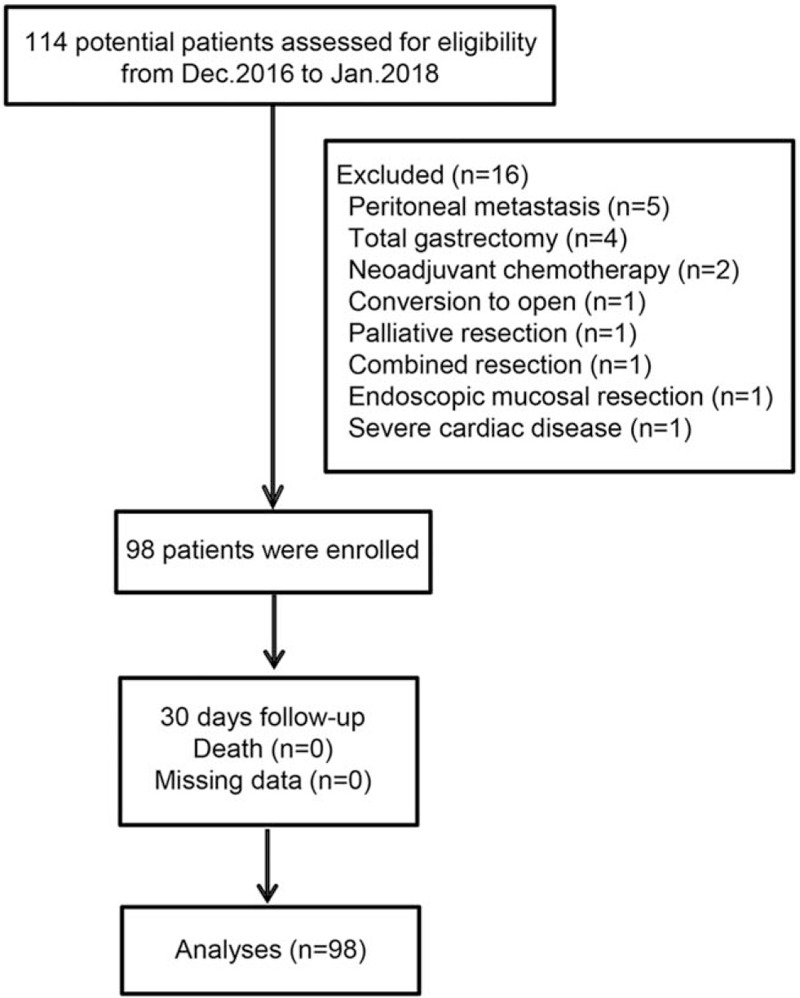
Flow chart.

A total of 114 patients were assessed for eligibility from December 2016 to January 2018, and 98 patients were finally enrolled. All patients were followed up 30 days after the operation (Fig. 1).

The subjects comprised 60.2% males (59 of 98 subjects) and 39.8% females (39 of 98 subjects). Their average age was 50.4 years. The mean operation time was 241.1 min, and the mean intraoperative blood loss was 68.8 mL. According to pathological diagnosis, the subjects comprised 7.2% stage Tis patients (7 of 98 subjects), 50% stage I patients (48 of 98 subjects), 21.4% stage II patients (21 of 98 subjects), and 21.4 stage III patients (21 of 98 subjects) (Table 2).

**Table 2:** Demographic data and surgery-related data.

Characteristics	
Number of subjects	98
Age (yr)	
Mean	50.4
Median(IQR^∗^)	52 (43–59)
Sex	
Male (%)	59 (60.2)
Female (%)	39 (39.8)
BMI^†^ (kg/m^2^)	
Mean	22.5
Median (IQR)	22.5 (20.4–24.5)
ECOG PS^‡^	
0 (%)	92 (93.9)
1 (%)	6 (6.1)
ASA PS^§^	
I (%)	44 (44.9)
II (%)	54 (55.1)
Operation time (min)	
Mean	241.1
Median (IQR)	244 (200–270.8)
Intraoperative blood loss (ml)	
Mean	68.8
Median (IQR)	50 (37.5–100)
Tumor size (mm)	
Mean	20.2
Median (IQR)	15.5 (5–30)
Retrieved lymph node	
Mean	41.7
Median (IQR)	40.5 (30.75–50.25)
Pathological stage	
Tis (%)	7 (7.2)
I (%)	49 (50)
II (%)	21 (21.4)
III (%)	21 (21.4)

### 3.2 Safety

The incidence of post-operative complications and mortality in 30 days after surgery was 20.4%, 0%, respectively. A total of 28 complications were seen in 20 patients. Six complications, including anastomotic leak, intestinal fistula, intra-abdominal abscess, ileus, pulmonary, and wound problem occurred in 1 patient who had undergone reoperation. Two patients had intra-abdominal bleeding, and 2 patients had gastroparesis. We observed that the pulmonary complication rate was 1.0%, and other complications were classified as Clavien - Dindo grade I or II. Six patients needed to be readmitted after discharge without reoperation. No adverse events related to this study occurred (Table 3).

**Table 3:** The incidence of postoperative complications and mortality in 30 days.

Postoperative complications	No.(n = 98)	Rate
Total cases^∗^	20	20.4%
Anastomotic Leak	1	1.0%
Intestinal fistula	1	1.0%
Ileus	7	7.1%
Gastroparesis	2	2.0%
Intra-abdominal bleeding	2	2.0%
Intraluminal bleeding	3	3.0%
Intra-abdominal abscess	3	3.0%
Lymphatic leakage	3	3.0%
Acute urinary retention	3	1.0%
Wound problem	1	1.0%
Pulmonary	1	1.0%
Cardiac	1	1.0%
Mortality	0	0%
Clavien -Dindo Classification		
I	5	5.1%
II	16	16.3%
IIIa	4	4.1%
IIIb	3	3.0%
IV	0	0%
V	0	0%
Readmission^†^	6	6.1%
Ileus	3	3.1%
Gastroparesis	2	2%
Cardiac	1	1%

### 3.3 Efficacy

#### 3.3.1 Recovery time and short-term recovery outcomes

Recovery time was defined as post-operative stay and rehabilitative rate on POD-4. We found that the median post-operative stay was 6.0 days, interquartile ranging from 3.0 to 7.0 days. Patients who satisfied rehabilitative and discharge criteria were considered rehabilitated and discharged, and the rehabilitative rate on POD-4 was 78% (Table 4).

**Table 4:** Recovery time and short-term recovery outcomes.

Variable	Value
Recovery time	
Postoperative stay (d)	
Mean	7.0
Median (IQR^∗^)	6.0 (3.0–7.0)
Rehabilitative rate on POD^†^-4	78%
Short-term recovery outcomes	
First ambulation time (d)	1.0
First flatus time (d)	2.4
Oral liquid diet recovery time (d)	3.0
Oral half flow diet recovery time (d)	4.5
Drainage removal time (d)	4.4

### 3.4 Post-operative pain score

**Figure 2:**
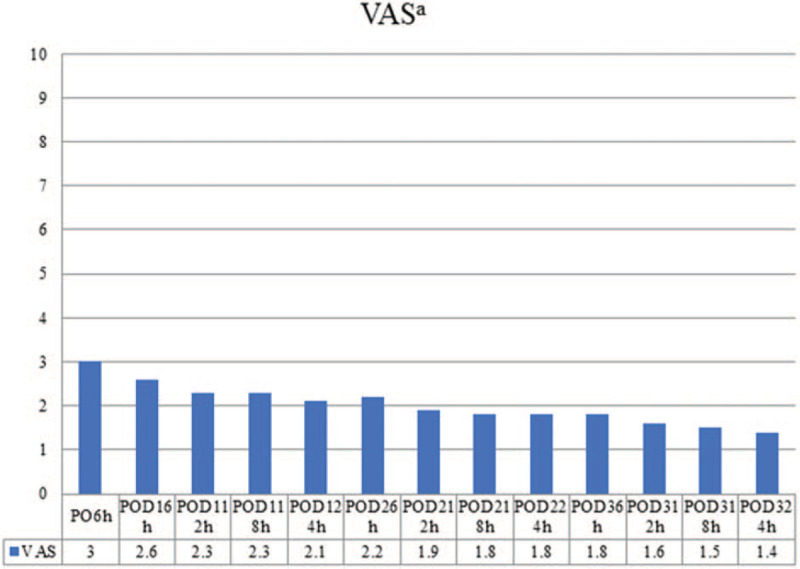
Post-operative pain score a VAS Visual analogue scale, POD Post-operative day.

We used the VAS score scale to evaluate post-operative pain on PODs 0–3. The first evaluation was 6 hours after surgery and repeated every 12 hours on PODs 1 to 3. Patients’ mean first VAS score was 3, and it reduced gradually to 1.4 on POD-3 (Fig. 2).

### 3.5 The compliance rate of the ERAS program

The compliance rates for the preoperative and intraoperative ERAS program were 100%. All the patients had no nasal gastric tube, or it was removed within 6 hours postoperatively. Five patients had a nasal gastric tube placed postoperatively. The urinary catheter was removed within 6 hours postoperatively in 96% of the patients, and 94% of the patients started to sip water after awakening from anesthesia. On POD 1, 86% of the patients started oral intake of clear water, and 89% of the patients began ambulation with assistance. On POD 2, 70% of the patients were tolerating a liquid diet, and 86% of patients could ambulate with assistance (1000–1500 meters). On POD 3, 60% of the patients tolerated a semi-fluid diet, and 85% of the patients could ambulate with assistance over 1500 meters. Only 20% percent of the patients needed additional anesthesia like intravenous NASID's and analgesics such as tramadol hydrochloride and opioid drugs postoperatively (Table 5).

**Table 5:** The compliance rate of ERAS protocol.

ERAS protocol	Compliance rate(%)
Preoperative	
Preoperative counseling and education about ERAS	100
No mechanical bowel preparation	100
Maintain a normal oral diet until midnight before surgery	100
Oral carbohydrate solution up to 2 h before surgery	100
Intraoperative	
Laparoscopic surgery and incision < 7cm	100
Surgical site NSAIDs infiltration	100
Antithrombotic prophylaxis and thermostasis	100
Postoperative	
Additional anesthesia and analgesics^∗^	20
No nasal gastric tube or removed within 6 h postoperatively^†^	100
Abdominal drainages removed within 72 h postoperatively	59
Urinary catheter removed within 6 h postoperatively	96
Sips of water when awake post operation	94
Oral clear water on POD-1	86
Oral liquid diet on POD-2	70
Oral semi-fluid diet on POD-3	60
Ambulation with assistance (500 - 1000 m) on POD-1	89
Ambulation with assistance (1000 - 1500 m) on POD-2	86
Ambulation with assistance (over 1500 m) on POD-3	85

## 4 Discussion

This prospective cohort study showed that the ERAS program is safe and effective for gastric cancer patients after LDG. Compared to the results of our institution in the CLASS-01 trial, the ERAS program is an optimal way to reduce post-operative hospital stay from 9.0 days to 6.0 days for gastric cancer patients after LDG.

A recent RCT by Kang et al^[22]^ reported that the ERAS group patients undergoing totally laparoscopic distal gastrectomy had a higher recovery rate, shorter recovery time, and less pain than the conventional surgery. However, several limitations weakened clinical significance. In this trial, from the point of view of the ethical consideration, the conventional group contained some of the ERAS protocols including no bowel preparation and no nasogastric tube insertion. Accordingly, proper randomization and allocation could not be achieved. Besides, double-blinding is difficult to be performed. Thus, a prospective cohort study would be an alternative to clarify this issue. In this cohort study enrolled 98 patients, the incidence of post-operative complications is 20.4% and is consistent with other multicenter studies ranging from 3.7% to 24.2%.^[5–7,23]^ Seven grade III complications were observed. However, 4 in 7 grade III complications occurred in 1 patient who had undergone reoperation. The surprising finding was that the pulmonary complication rate in this study was significantly lower than the results of our institution in the CLASS-01 trial (1% vs 5.6%).^[8]^ This reduction may be related to the effective gastric tube management and early, goal-oriented ambulation in the ERAS program.^[24]^ Meanwhile, we observed six readmissions, and 5 in 6 were because of complications like ileus and gastroparesis. Unlike the multimodal primary care including retail healthcare, concierge and direct care, and home-based diagnostics and care in the United States, the hierarchical medical system in China is still under construction.^[25]^ Therefore, patients in China tend to attend a general hospital to treat complications like ileus and gastroparesis rather than attend to primary care. Besides, the cooperation between primary care and the ERAS Society in China is still insufficient. In Alberta, Canada, the state health care service worked with the ERAS Society to implement ERAS. The results were promising, with shorter stays, lower complications and readmission rate.^[26]^ In the future, the complication and readmission rate might be reduced through the construction of the hierarchical medical system in China.

The primary efficacy assessment was the recovery time. Prior to this study, a randomized controlled trial was conducted to compare laparoscopic gastrectomy and open distal gastrectomy for advanced gastric cancer.^[8]^ With similar demographic data and surgery-related data, the present study subjects had a shorter median post-operative stay, compared to the results of our institution of the LDG group in the CLASS-01 trial (6.0 vs 9.0 days). Additionally, 78% of subjects satisfied rehabilitative and discharge criteria on POD-4. The reduction of post-operative stay may be attributed to the enhanced recovery of bowel function and effectiveness of multimodal analgesia which consisted of surgical site infiltration of NSAIDs. First flatus time was used to predict the bowel recovery time. Patients in this study had shorter first flatus time, liquid recovery time, and first ambulation time respectively, compared with the results of our institution in the CLASS-01 trial (2.4 vs 3.5 days, 3.0 vs 5.5 days, 1.0 vs 2.3 days). These results suggested that the ERAS program may enhance bowel function recovery for its short fasting, early oral feeding, and early, goal-oriented ambulation protocols. Similar results had been observed in the ERAS program of bariatric surgery and colorectal surgery.^[27–34]^ Although early oral feeding is 1 of the impact factors that lead to early recovery of bowel function, the best protocol to start oral feeding is still unknown. A significant decrease of first flatus time was observed in the ERAS group of bariatric surgery (9.2 ± 3.4 h vs 16.6 ± 8.1 h, *P* = .008).^[27]^ Moreover, in the ERAS program of colorectal surgery, shorter first flatus time was shown in the ERAS program that offered a carbohydrate-loaded drink on the day after surgery,^[28,29,31,34]^ compared to other ERAS program that fasting or offering water only on the day after surgery^[30,32,33]^ (30.6 h [21.6–38.4 h] vs. 53.94 h [50.4–58 h]). Similar results were also observed in an RCT focusing on patients under McKeown minimally invasive esophagectomy.^[35]^ The decrease in first flatus time by early oral feeding might be caused by activating of normal digestive reflexes. Still, additional studies should be developed to focus on the optimal early oral feeding program after patients undergoing laparoscopic gastrectomy. Conventional midnight fasting is related to insulin resistance and discomfort after surgery.^[10]^ Actually, the intake of clear fluids up to 2 hours before surgery is safe, according to the ASA.^[36]^ Furthermore, consensus guidelines report that early oral feeding can be attributed to faster bowel function recovery without increasing the risk of fistulas.^[10]^ In this study, we provided a feasible and effective early ambulation protocol with an accurate ambulation training goal rather than an ambiguous protocol like continue and encourage ambulation.^[22]^ With this early, goal-oriented ambulation protocol, unwanted effects and delayed resumption of gut function may be avoided.^[24,37]^

The pain control protocol in this study is multimodal analgesia. We used multimodal analgesia including surgical site infiltration of NSAIDs rather than epidural analgesia or intravenous analgesia which was recommended by ERAS Society.^[10]^ Because NSAIDs are commonly used as a component of multimodal analgesia to assure better analgesia and reduce the dose of opioids.^[38,39]^ Multimodal analgesia including surgical site infiltration of NSAIDs might also achieve better analgesia and reduction of opioids used. The patient's first mean VAS score is 3 and reduced gradually to 1.4 on POD-3. VAS score lower than 3 is defined as mild, annoying pain, which is tolerable and acceptable. Besides, only 1 patient needed additional opioid drugs after the operation in this study. From this point of view, the multimodal analgesia which consisted of surgical site infiltration of NSAIDs might be effective.

The compliance rate of the ERAS program is an indicator of the feasibility of the program. Besides, a study of 2352 patients with colorectal cancer revealed the importance of compliance. With better compliance, the length of hospital stay and the complication rate could be reduced.^[39]^ In this study, preoperative and intraoperative parts of the ERAS program are resolutely implemented by the multidisciplinary team to ensure better physical condition for patients. And with the variation of the patient's physical and mental condition after being traumatized, we implemented an individualized post-operative ERAS program. Still, more than one-half of patients can tolerate early intake of liquid or semi-liquid, and over 80% of patients ambulate early. From this perspective, early intake of liquid or semi-liquid and early, goal-oriented ambulation are feasible. However, the compliance rate for removing drainage within 72 hours is 59%, which may due to the varied operative and physical conditions of patients.

This study has several limitations. First, this study had a relatively small sample size. Therefore, the findings need to be interpreted with caution, and no definitive conclusions can be reached at this time. Nevertheless, the trends observed in this study are similar to other studies of abdominal surgery with the ERAS program including some randomized controlled ones.^[22,40,41]^ Second, multimodal analgesia which consisted of surgical site infiltration of NSAIDs is used to replace epidural and intravenous analgesia. The outcome of the VAS score and the reduction of opioids are satisfactory. Third, the drain is still placed during the operation to the early detection of anastomotic leakage in this study, and that may result in longer hospital stays and higher post-operative complication rate.^[10]^ Fourth, this study is performed in a single institution, and a multicenter approach may be needed to generalize the results to a larger population.

In summary, the present study suggests that the combination of the ERAS program and LDG might be an effective way to reduce recovery time and hospital stay without compromising safety. And this combination might be optimized perioperative management for gastric cancer patients after LDG.

## Acknowledgments

We thank Xinhua Chen for help with preparing the manuscript. We thank Jaime Ruiz-Tovar, MD, Ph.D., for providing the first flatus time data of their prospective study.

## Author contributions

**Conceptualization:** Hao Liu.

**Data curation:** Yanrui Liang, Hao Liu, Li Zhen Nurse, Mingli Zhao, Yanfeng Hu.

**Formal analysis:** Yanrui Liang, Hao Liu, Li Zhen Nurse, Kexuan Liu, Guoxin Li.

**Funding acquisition:** Kexuan Liu, Guoxin Li.

**Investigation:** Yanrui Liang, Hao Liu, Li Zhen Nurse, Yu Zhu, Mingli Zhao, Yanfeng Hu, Jiang Yu, Cai Li, Kexuan Liu, Guoxin Li.

**Methodology:** Hao Liu, Li Zhen Nurse, Yu Zhu, Jiang Yu, Cai Li, Kexuan Liu, Guoxin Li.

**Project administration:** Jiang Yu, Kexuan Liu, Guoxin Li.

**Resources:** Li Zhen Nurse, Kexuan Liu, Guoxin Li.

**Software:** Yanrui Liang, Yu Zhu, Mingli Zhao, Yanfeng Hu, Cai Li.

**Supervision:** Kexuan Liu, Guoxin Li.

**Validation:** Cai Li, Guoxin Li.

**Visualization:** Yanrui Liang.

**Writing – original draft:** Yanrui Liang, Hao Liu, Li Zhen Nurse.

**Writing – review & editing:** Yanrui Liang, Hao Liu, Kexuan Liu, Guoxin Li.

## Supplementary Material

Supplemental Digital Content
