# Supplementary material for: Enhanced recovery after surgery for laparoscopic gastrectomy in gastric cancer: A prospective study
Source: Medicine (Baltimore). 2021 Feb 19;100(7):e24267. doi: 10.1097/MD.0000000000024267 (PMC7899858; doi:10.1097/MD.0000000000024267)
Supplement: Supplemental Digital Content [file medi-100-e24267-s001.doc]

Supplemental Digital Content 1

**Table S1** Inclusion, exclusion, and withdrawal criteria

| Inclusion criteria | Exclusion criteria | Withdrawal criteria |
| --- | --- | --- |
| 18 years < age < 75 years | Severe mental disorder | Distant metastasis |
| Pathologically proven primary gastric adenocarcinoma by endoscopic biopsy | Women of childbearing potential who were pregnant or breastfeeding | Curative resection through total gastrectomy |
| cT1-4a, N0-3, M0 at preoperative evaluation according to the AJCC^c^ Cancer Staging Manual Seventh Edition | History of previous upper abdominal surgery | Conversion to open surgery |
| Expected curative resection through laparoscopic distal gastrectomy | History of previous neoadjuvant chemotherapy, radiotherapy or clinical trial treatment within three months | Combined organ resection |
| No history of prior/other malignancies within the past five years prior to enrollment with the exception of basal cell carcinoma  ECOG PS^a^ 0 or 1 | History of other malignant diseases within the past five years  Contraindications for surgery including active systemic infections, coagulation disorders | Inability to undergo surgery or anesthesia for the changing illness state  Intraoperative bleeding over 400 ml or transfusion |
| ASA PS^b^ I or II | Other major medical illnesses of the cardiovascular, respiratory, or immune system | Intraoperative bleeding over 400 ml |
| Adequate organ function  Informed consent | History of myocardial infarction or cerebrovascular accident within the past six months  Emergency surgery due to complication (bleeding, obstruction, or perforation) caused by gastric cancer | Required to withdraw by patients |

a ECOG PS Eastern Cooperative Oncology Group performance status, b ASA PS American Society of Anesthesiologists physical status, c AJCC American Joint Committee on Cancer
